# Development of the nursing artificial intelligence readiness scale for nursing students: a validity and reliability study

**DOI:** 10.1186/s12912-026-05054-4

**Published:** 2026-07-16

**Authors:** Sümeyye Akçoban, Gülay Koca, Soner Berşe

**Affiliations:** 1 Department of Health Services, Kırıkhan Vocational School, Hatay Mustafa Kemal University, Hatay, Turkey; 2 Department of Nursing, Faculty of Health Sciences, Gaziantep University, Şehitkamil, Gaziantep, 27310 Turkey

**Keywords:** Artificial intelligence, Nursing students, Readiness, Scale development, Validity, Reliability

## Abstract

**Background:**

The rapid integration of artificial intelligence (AI) into healthcare has amplified the need for nurses who can engage with AI-supported systems safely and effectively. Assessing nursing students’ AI readiness through a psychometrically sound instrument is essential for guiding curriculum design and targeted educational interventions. This study aimed to develop and psychometrically evaluate the Nursing Artificial Intelligence Readiness Scale (NAIRS) for nursing students.

**Methods:**

This methodological scale development study was conducted with 416 nursing students enrolled during the 2025–2026 academic year at a public university in Türkiye. To avoid conducting exploratory and confirmatory factor analyses on the same dataset, the full sample was randomly split into two independent subsamples (EFA: *n* = 200; CFA: *n* = 216). The two subsamples were comparable in key demographic and AI-related characteristics. An initial 40-item draft was generated from the literature and reviewed by 10 faculty experts using a content validity evaluation; the mean CVR was 0.88. Following expert review, five items were removed, reducing the pool to 35 items. The revised draft was then pilot tested with 50 nursing students to assess item clarity, readability, comprehensibility, and administration flow, and minor revisions were made prior to the main application. The final 20-item scale was structured across four theoretical domains: Knowledge/Awareness, Willingness to Use AI, Self-efficacy, and Ethical Awareness. Construct validity was examined using EFA (minres extraction; Promax rotation) and first-order CFA (ML estimation) with multiple model fit indices. Convergent and discriminant validity were assessed using CR/AVE and the Fornell–Larcker criterion, respectively. Internal consistency was evaluated with Cronbach’s alpha in both subsamples, and temporal stability was tested via a 2-week test–retest application in a subgroup (*n* = 45) using ICC (two-way random, single measures).

**Results:**

The final scale demonstrated a four-factor structure explaining 52.08% of the total variance, with factor loadings ranging from 0.351 to 0.909. CFA supported the proposed model with good fit (χ²/df = 1.426, CFI = 0.971, TLI = 0.967, GFI = 0.910, RMSEA = 0.044, SRMR = 0.049). Internal consistency was high: subscale alphas ranged 0.801–0.828 (EFA sample) and 0.858–0.902 (CFA sample), and the total scale alpha was 0.911. Test–retest reliability indicated strong stability, with ICC values ranging 0.896–0.963 across subscales and 0.952 for the total scale.

**Conclusions:**

The findings provide initial evidence that the NAIRS is a valid and reliable instrument for assessing nursing students’ readiness for artificial intelligence across knowledge/awareness, willingness to use AI, self-efficacy, and ethical awareness domains. The scale may be useful for educational needs assessment and curriculum planning in nursing education.

**Clinical trial number:**

Not applicable.

**Supplementary Information:**

The online version contains supplementary material available at https://doi.org/10.1186/s12912-026-05054-4.

## Contributions to the Literature

- This study provides a nursing-context–specific, psychometrically tested instrument to assess nursing students’ readiness for AI technologies using a rigorous EFA–CFA cross-validation approach (EFA *n* = 200; CFA *n* = 216).
- The scale conceptualizes AI readiness as a multidimensional construct comprising knowledge/awareness, willingness to use AI, self-efficacy, and ethical awareness.
- The findings provide psychometric evidence supporting the use of the instrument in educational needs assessment, curriculum evaluation, and the planning of targeted AI-related training in nursing education.

## Introduction

In the twenty-first century, the widespread adoption of computers and the internet has transformed the technology and information sectors into innovation-driven fields. Subsequently, artificial intelligence applications have rapidly become an indispensable part of daily life [1]. This transformation process has necessitated that individuals and institutions continuously adapt and embrace innovation as a strategic requirement [2]. In particular, digitalization and data-driven decision-making approaches in clinical processes related to healthcare delivery have gained momentum globally. AI-supported applications have become increasingly prominent in diagnosis, monitoring, risk classification, and clinical decision support [3].

The increasing use of artificial intelligence in healthcare systems directly affects nursing practice. Nursing is a critical stakeholder in the design, implementation, and evaluation of AI-based systems in areas such as clinical workflows, care planning, patient safety, and continuity of care [4]. Indeed, the literature on AI applications in nursing indicates that AI-based technologies are rapidly diversifying in nursing practice; however, significant needs persist regarding education, acceptance, workflow integration, and ethical/professional role ambiguity in the transition to practice. In this context, nurses’ knowledge, skills, and attitudes toward AI are considered prerequisites for safe and effective use in clinical practice [5, 6].

For sustainable management of this transformation, it is important to assess not only AI literacy and technological competencies but also students’ attitudes toward AI in nursing education. A study conducted with nursing students reported that students generally exhibited positive attitudes toward AI; however, these attitudes should be considered together with educational needs, intention to use, and anxiety levels [7]. Similarly, another study indicated that students’ readiness and acceptance levels for AI-based technologies were at a moderate level and that this needed to be supported through structured education [8].

Readiness is defined as a multidimensional construct encompassing an individual’s tendency to adopt and use new technology, perceived competence, and openness to change. In the technology readiness literature, this concept has been addressed within a theoretical framework measuring individuals’ propensity to embrace new technologies [2]. In the context of AI, readiness extends beyond the willingness to use and encompasses understanding clinical roles, benefit–risk assessment, ethical sensitivity, and self-efficacy. The MAIRS-MS, developed in this direction, is one of the instruments providing validity and reliability evidence for measuring readiness for medical AI [9]. Current studies show that readiness for AI among nursing students is associated with anxiety, literacy, and attitude variables, and that the combined assessment of these constructs is important for educational planning [10, 11].

However, when the literature is examined, two key limitations emerge: (i) existing measures of AI among nursing students often focus on a single construct (e.g., readiness or acceptance alone) or rely on adaptations of instruments developed for other professional groups; (ii) the number of nursing-specific instruments that comprehensively assess multiple dimensions of AI readiness within the same psychometric framework remains limited [4, 8, 9]. Therefore, there is a need for a valid and reliable assessment tool that comprehensively assesses multiple dimensions of AI readiness aligned with nursing education outcomes. The aim of this study was to develop and psychometrically evaluate the Nursing Artificial Intelligence Readiness Scale (NAIRS) for nursing students.

## Methods

### Study design

This methodological study was conducted to develop and psychometrically evaluate a valid and reliable instrument for assessing nursing students’ readiness for artificial intelligence (AI) technologies. The study followed established scale development procedures, including item generation, expert review for content validity, pilot testing, item analysis, exploratory factor analysis, confirmatory factor analysis, and reliability testing. The research was conducted following the scale development steps recommended by DeVellis (2017) and Tavşancıl (2018) [12, 13].

### Study population and sample

The study population comprised all students enrolled in the nursing department of a public university during the 2025–2026 academic year. A total of 416 nursing students who voluntarily agreed to participate and completed the data collection forms in full were included. In scale development studies, a sample size of at least 5–10 times the number of items is recommended [13, 14]. Because the initial draft scale consisted of 40 items, a minimum of 200–400 participants was targeted, and this criterion was met.

Considering that conducting exploratory factor analysis (EFA) and confirmatory factor analysis (CFA) on the same sample is methodologically inappropriate [15, 16], the full dataset was randomly divided into two subgroups: 200 participants for EFA and 216 participants for CFA. After data collection was completed, participants were assigned a random number using a computer-generated randomization procedure and sorted accordingly. The first 200 cases were allocated to the EFA subsample and the remaining 216 cases to the CFA subsample, ensuring that each participant had an equal probability of being assigned to either group. This split allowed the structure to be verified in an independent sample through cross-validation. The comparability of the two subsamples was examined using an independent-samples t-test for age and chi-square tests for sex, academic year, previous AI training, and frequency of AI use; no statistically significant differences were found (all *p* > .05). Additionally, to evaluate test–retest reliability, the scale was re-administered after a two-week interval to 45 randomly selected participants from the EFA subsample. For the test–retest procedure, participants from the EFA subsample were assigned random numbers using the same computer-generated randomization approach, and 45 students were selected for re-assessment. All selected participants completed the second administration after the two-week interval.

### Data collection tools

#### Item generation, content validity, and pilot testing

An initial item pool was generated based on a review of the literature on artificial intelligence readiness, technology readiness, digital health literacy, and the use of AI in nursing and healthcare education. The preliminary draft consisted of 40 items representing four theoretically defined domains: During item generation, particular attention was given to ensuring that the items reflected nursing-specific educational and clinical contexts rather than general technology acceptance. The initial pool intentionally included overlapping and conceptually similar items to allow subsequent refinement during expert evaluation and psychometric testing. Examples of the initial items addressed topics such as understanding the basic principles of AI, confidence in using AI-supported systems, willingness to incorporate AI into future nursing practice, and awareness of ethical issues related to AI-assisted decision-making. To assess content validity, the draft items were reviewed by 10 faculty experts specializing in nursing sciences and measurement/evaluation. The experts evaluated each item in terms of relevance, clarity, comprehensibility, and representativeness using a three-point rating scale. Based on these evaluations, the mean content validity ratio (CVR) was 0.88 [17]. Following expert review, five items were removed from the initial pool. These items were excluded because they were judged to be conceptually redundant with other items, insufficiently representative of the target construct, or ambiguously worded. Several remaining items were revised to improve clarity, linguistic precision, and consistency with the theoretical definitions of the four domains. Following expert review, the revised draft scale was pilot tested with 50 nursing students to assess item clarity, comprehensibility, readability, and the overall administration process. The pilot participants were also invited to provide qualitative feedback regarding item wording, interpretation, and ease of completion. Although no items were removed during the pilot phase, minor wording and formatting revisions were made to improve readability and reduce potential misunderstandings. Based on feedback obtained during the pilot application, minor wording and formatting revisions were made before the main data collection.

#### Personal information form

This form was prepared by the researchers to collect information regarding participants’ age, sex, academic year, prior AI-related training, and frequency of AI use.

#### Nursing Artificial Intelligence Readiness Scale (NAIRS)

The draft scale was developed as a total of 40 items across four theoretical dimensions through a comprehensive review of the relevant literature, evaluation of existing scales, and expert consultation: (a) Knowledge/Awareness (10 items), (b) Willingness to Use AI (10 items), (c) Self-efficacy (10 items), and (d) Ethical Awareness (10 items). Items were rated on a 5-point Likert scale (1 = Strongly Disagree, 2 = Disagree, 3 = Undecided, 4 = Agree, 5 = Strongly Agree). Total scores on the final 20-item scale range from 20 to 100, with higher scores indicating higher AI readiness.

### Data collection

Data were collected online during the 2025–2026 fall semester. Participants were provided with detailed information about the purpose and procedures of the study, and voluntary informed consent was obtained. Only students who provided informed consent were included. Ethical approval was obtained from the relevant university’s ethics committee. The final version used in the main application was established after the completion of expert review and pilot testing procedures.

### Data analysis

Data were analyzed using descriptive statistics, item analysis, exploratory factor analysis, confirmatory factor analysis, convergent and discriminant validity analyses, and reliability analyses. Statistical analyses were performed in Python 3.10 using the factor_analyzer, semopy, scipy, and pandas libraries.

#### Item analysis

Item discrimination was evaluated using three criteria: (1) corrected item–total correlation (*r* ≥ .30 cutoff [18]), (2) differences between the lower 27% and upper 27% groups (independent samples t-test), and (3) changes in Cronbach’s alpha if an item was deleted. Items with corrected item–total correlations below 0.30, those showing no significant difference in upper–lower group comparisons, or those whose removal increased the alpha value were eliminated.

#### Construct validity

Before EFA, the suitability of the data for factor analysis was examined using the Kaiser–Meyer–Olkin (KMO) measure of sampling adequacy and Bartlett’s test of sphericity. The number of factors was determined using parallel analysis [19], the Kaiser criterion (eigenvalue > 1), and inspection of the scree plot. The minimum residual (minres/ordinary least squares) extraction method was used, and Promax (oblique) rotation was selected. Promax rotation allows the correlations between factors that are theoretically expected to be related to be freely estimated [20]. Items were primarily evaluated using loadings of 0.40 or higher on their primary factor and absence of cross-loadings above 0.32 on more than one factor; however, items with loadings between 0.35 and 0.39 were considered for retention when they were theoretically relevant, did not show problematic cross-loadings, and were supported by acceptable corrected item–total correlations and satisfactory CFA loadings [21].

To confirm the identified structure, first-order CFA was performed on the independent CFA subsample (*n* = 216) using maximum likelihood (ML) estimation. Prior to CFA, the dataset was screened for missing data and distributional assumptions. Only questionnaires that were completed in full were included in the analyses; therefore, no missing data were present in the final dataset and no imputation procedures were required. The distributional characteristics of the observed variables were examined using skewness and kurtosis statistics. Skewness values ranged from − 0.94 to 0.12 and kurtosis values ranged from − 0.82 to 0.29, indicating no severe deviation from normality [22] and the results supported the use of maximum likelihood estimation. In addition, item distributions were examined for extreme asymmetry and potential floor or ceiling effects, and no problematic patterns were identified. These findings further supported the appropriateness of maximum likelihood estimation for the CFA analyses. Model fit was evaluated using multiple fit indices: χ²/df ratio, Comparative Fit Index (CFI), Tucker–Lewis Index (TLI), Goodness-of-Fit Index (GFI), Adjusted Goodness-of-Fit Index (AGFI), Normed Fit Index (NFI), Root Mean Square Error of Approximation (RMSEA), and Standardized Root Mean Square Residual (SRMR). Reference values from the relevant literature were used as acceptable and good fit criteria [23–25].

#### Convergent and discriminant validity

Convergent validity was assessed using Composite Reliability (CR ≥ 0.70) and Average Variance Extracted (AVE ≥ 0.50). Discriminant validity was evaluated using the Fornell–Larcker criterion, requiring each subscale’s √AVE to exceed its correlations with other subscales [26, 27].

#### Reliability

Internal consistency reliability was calculated using Cronbach’s alpha coefficients separately in both subsamples (EFA and CFA). Temporal stability was assessed in the 45-participant subgroup using the Intraclass Correlation Coefficient (ICC; two-way random, single measures) and Pearson correlation coefficients from two measurements taken two weeks apart. Paired-samples t-tests were used to compare the means between the two measurements. The classification proposed by Koo and Li (2016) was used for interpreting ICC values: <0.50 poor, 0.50–0.75 moderate, 0.75–0.90 good, > 0.90 excellent [28].

### Ethics approval and consent to participate

Ethical approval was obtained from the Non-Interventional Research Ethics Committee of Gaziantep University (Session Date: 28/05/2025; Decision No: 2025/179). The study was conducted in accordance with the ethical principles of the Declaration of Helsinki. All participants provided informed consent prior to participation.

## Results

### Content validity and pilot testing

Before the main application, the draft items were reviewed by 10 faculty experts specializing in nursing sciences and measurement/evaluation. Using Lawshe’s (1975) three-point format, experts rated each item as essential, useful but not essential, or not necessary. Item-level content validity ratios were calculated and averaged across items; the mean CVR was 0.88 [17]. In addition, the experts provided qualitative feedback regarding clarity, comprehensibility, and representativeness, and wording revisions were made accordingly. Following revisions based on expert feedback, the scale was pilot tested with 50 nursing students, and minor wording and formatting changes were made prior to the main administration. The 50 students who participated in the pilot testing were not included in the main psychometric analyses.

### Participant characteristics

A total of 416 nursing students participated in the study. The mean age of the participants was 20.95 ± 3.59 (min = 18, max = 48). Of the participants, 53.4% (*n* = 222) were female and 46.6% (*n* = 194) were male. Regarding academic year distribution, first-year students constituted the largest group (53.8%, *n* = 224). Of the participants, 32.5% (*n* = 135) reported having received AI-related training, while 67.5% (*n* = 281) had not. Regarding frequency of AI use, 34.6% (*n* = 144) reported “sometimes,” 26.9% (*n* = 112) “often,” and 15.6% (*n* = 65) “very often” (Table 1).

**Table 1:** Sociodemographic characteristics of the participants (*n* = 416)

Variable	Category	*n*	%	Mean ± SD
Sex	Female	222	53.4	
	Male	194	46.6	
Academic Year	1st year	224	53.8	
	2nd year	59	14.2	
	3rd year	73	17.5	
	4th year	60	14.4	
AI Training	Yes	135	32.5	
	No	281	67.5	
Frequency of AI Use	Never	23	5.5	
	Rarely	72	17.3	
	Sometimes	144	34.6	
	Often	112	26.9	
	Very often	65	15.6	
Age				20.95 ± 3.59

Table 1 presents the sociodemographic characteristics of the participants. The EFA and CFA subsamples did not differ significantly in terms of age (t = − 0.516, *p* = .606), sex (χ² = 0.121, *p* = .728), academic year (χ² = 2.127, *p* = .546), previous AI training (χ² = 0.112, *p* = .738), or frequency of AI use (χ² = 4.609, *p* = .330).

### Item analysis

Starting from an initial pool of 40 items, 5 items were removed during the expert review phase based on content validity evaluations, reducing the pool to 35 items. The 35-item version was then pilot tested with 50 nursing students; no items were removed at this stage, but minor wording adjustments were made. Subsequently, item reduction procedures were conducted using the EFA subsample (*n* = 200). Items were evaluated according to multiple criteria, including corrected item–total correlations, factor loadings, cross-loadings, communality values, and theoretical consistency with the intended construct. Fifteen items were removed because they demonstrated corrected item–total correlations below the recommended threshold, substantial cross-loadings on multiple factors, low communality values, or insufficient conceptual fit with the emerging factor structure, resulting in the final 20-item scale. The corrected item–total correlations of the 20 items in the final scale ranged from 0.361 to 0.614, all exceeding the 0.30 cutoff [18]. The differences between the lower 27% and upper 27% groups were statistically significant for all items (*p* < .001). Removal of any single item did not increase the total Cronbach’s alpha coefficient (0.880). These findings indicate that all items in the final scale have adequate discriminative power and make meaningful contributions to the scale.

### Exploratory factor analysis

Before EFA, the suitability of the data for factor analysis was assessed. The Kaiser–Meyer–Olkin (KMO) measure of sampling adequacy was 0.858, indicating that the sample size was “good” for factor analysis [29]. Bartlett’s test of sphericity was statistically significant (χ²(190) = 1742.04, *p* < 0.001), confirming that the correlation matrix was not an identity matrix and that factor analysis was applicable.

When parallel analysis and the Kaiser criterion (eigenvalue > 1) were evaluated together, a four-factor structure was identified as appropriate. The eigenvalues of the first four factors were 6.313, 2.417, 1.757, and 1.595, respectively, while the fifth factor’s eigenvalue (0.937) was below 1. According to the pattern matrix obtained with Promax rotation, 20 items clustered under four factors, explaining 52.08% of the total variance. This value meets the 40–60% range expected in the social sciences [13]. Primary factor loadings ranged from 0.351 to 0.909. Although a small number of items showed relatively modest loadings, these items were retained because they were conceptually relevant, did not exhibit problematic cross-loadings, and demonstrated acceptable corrected item–total correlations together with satisfactory standardized loadings in the CFA model (Table 4). In particular, items M30 and M38 showed relatively lower factor loadings (0.351 and 0.386, respectively) and lower communality values compared with the other items. However, both items were retained because they did not exhibit problematic cross-loadings, demonstrated acceptable corrected item–total correlations (M30 = 0.461; M38 = 0.361), and loaded satisfactorily on their respective latent factors in the CFA model (M30 = 0.691; M38 = 0.798). Furthermore, these items represented conceptually important aspects of the self-efficacy and ethical awareness domains that were not fully captured by the remaining items. Removing either item did not result in a meaningful improvement in internal consistency reliability or overall model fit. Therefore, both statistical evidence and theoretical considerations supported their retention in the final scale.

Table 2 shows that the four-factor structure is fully consistent with the theoretical framework. The five items under Factor 1 (Knowledge/Awareness; M1, M4, M6, M8, M9) measure knowledge of AI fundamentals and healthcare applications. The items under Factor 2 (Willingness to Use AI; M11, M12, M13, M15, M17) reflect willingness and attitudes toward using AI technologies. The Factor 3 (Self-efficacy) items (M21, M26, M28, M29, M30) measure self-confidence and perceived competence in using AI systems. The Factor 4 (Ethical Awareness) items (M31, M35, M38, M39, M40) assess awareness of ethical values in AI use.

**Table 2:** Pattern matrix from exploratory factor analysis with promax rotation (*n* = 200)

Item	Factor 1	Factor 2	Factor 3	Factor 4	h²
	(Knowledge)	(Willingness to Use AI)	(Self-efficacy)	(Ethics)	
M1	**0.812**	0.020	− 0.061	0.134	0.681
M4	**0.584**	0.026	0.025	− 0.049	0.345
M6	**0.460**	0.008	0.221	− 0.057	0.263
M8	**0.681**	0.105	0.069	− 0.017	0.480
M9	**0.849**	− 0.067	− 0.089	0.052	0.736
M11	− 0.065	**0.805**	0.111	− 0.068	0.668
M12	0.144	**0.703**	− 0.027	− 0.016	0.516
M13	0.051	**0.909**	− 0.121	− 0.104	0.854
M15	− 0.223	**0.530**	0.135	0.175	0.379
M17	0.173	**0.485**	− 0.017	− 0.068	0.270
M21	− 0.043	0.128	**0.773**	− 0.018	0.615
M26	− 0.015	0.030	**0.827**	− 0.040	0.686
M28	− 0.024	− 0.039	**0.871**	− 0.105	0.772
M29	0.064	− 0.085	**0.567**	0.061	0.337
M30	0.094	0.032	**0.351**	0.159	0.159
M31	− 0.103	0.104	0.049	**0.565**	0.343
M35	0.014	− 0.192	0.003	**0.823**	0.715
M38	0.040	0.237	− 0.125	**0.386**	0.222
M39	− 0.006	− 0.131	0.068	**0.853**	0.750
M40	0.143	− 0.036	− 0.059	**0.774**	0.624
**Eigenvalue**	**6.313**	**2.417**	**1.757**	**1.595**	
**Variance %**	**13.25**	**13.11**	**12.96**	**12.76**	
**Total variance explained (%)**	**52.08**				

Note. Factor loadings ≥ 0.35 are shown in bold. Extraction method: Minres; Rotation: Promax. KMO = 0.858; Bartlett χ²(190) = 1742.04, *p* < 0.001

The similar contribution of each factor to the total variance (12.76%–13.25%) indicates a balanced distribution of subscale weights within the scale. The total explained variance of 52.08% demonstrates that the scale adequately represents the construct. The moderate inter-factor correlations (0.22–0.51) obtained after Promax rotation support that the dimensions are related but distinguishable constructs.

### Confirmatory factor analysis

The four-factor structure obtained from EFA was tested through first-order CFA on the independent CFA subsample (*n* = 216). Model fit indices are presented in Table 3.

**Table 3:** Confirmatory factor analysis fit indices (*n* = 216)

Fit Index	Value	Acceptable Fit	Good Fit
χ²/sd	1.426	2–5	< 2
CFI	0.971	≥ 0.90	≥ 0.95
TLI	0.967	≥ 0.90	≥ 0.95
GFI	0.910	≥ 0.85	≥ 0.90
AGFI	0.896	≥ 0.80	≥ 0.90
NFI	0.910	≥ 0.90	≥ 0.95
RMSEA	0.044	≤ 0.08	≤ 0.05
SRMR	0.049	≤ 0.10	≤ 0.05

Note. Reference values: Hu & Bentler (1999), Kline (2016), Schermelleh-Engel et al. (2003)

Table 3 shows that the model demonstrated good fit to the data. The χ²/df ratio (1.426) being below 2 indicates excellent model fit [20]. CFI (0.971) and TLI (0.967) exceeded 0.95, reflecting good fit. GFI (0.910) was above the 0.90 threshold. AGFI (0.896) was at an acceptable level. NFI (0.910) exceeded the acceptable cutoff. Both RMSEA (0.044) and SRMR (0.049) were below 0.05, indicating good fit [23]. The simultaneous achievement of acceptable or good fit across all indices strongly supports the confirmation of the four-factor structure identified in EFA.

**Fig. 1:**
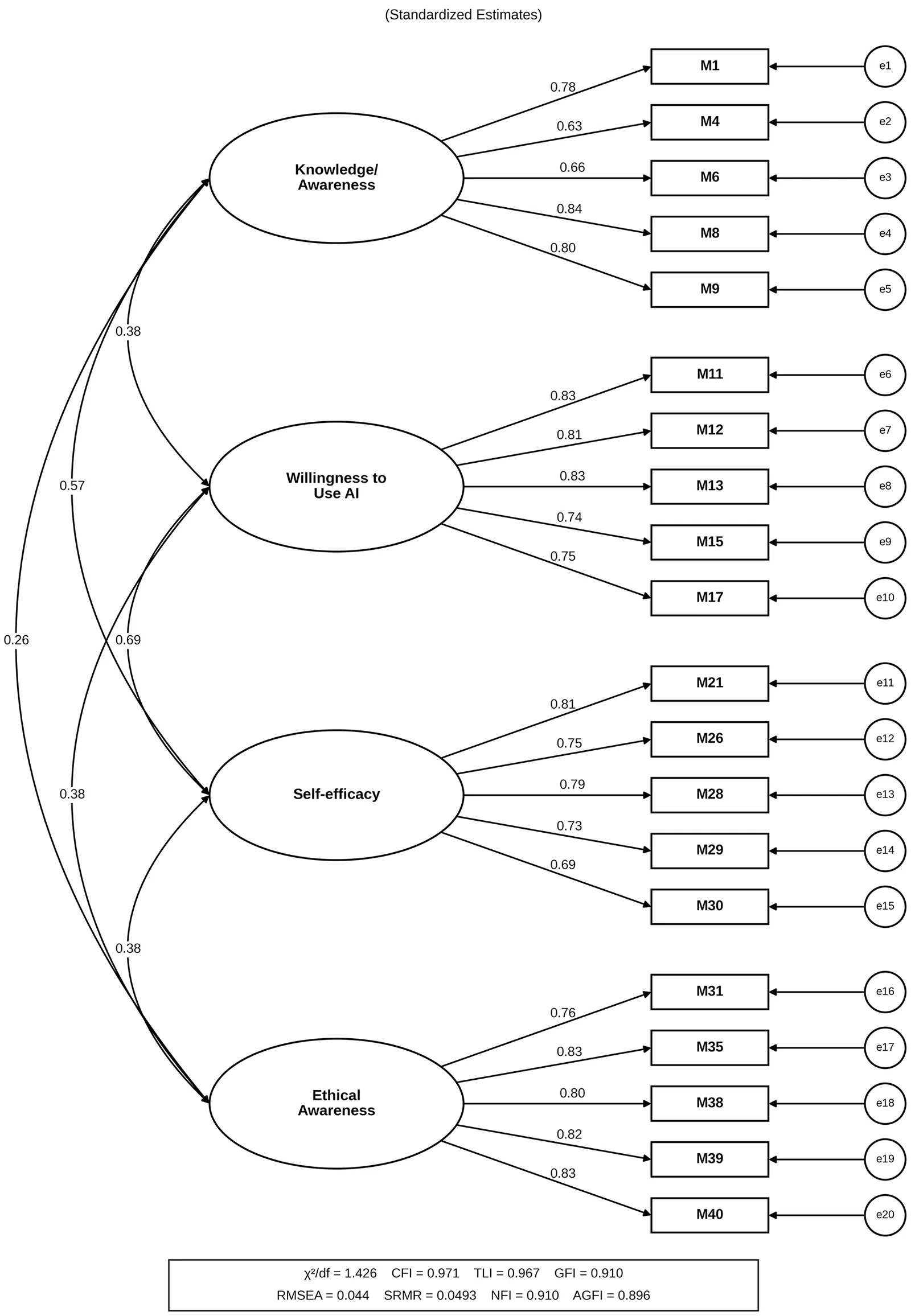
Confirmatory factor analysis path diagram (Standardized Estimates)

Standardized factor loadings ranged from 0.626 to 0.838 for Knowledge/Awareness, 0.738 to 0.832 for Willingness to Use AI, 0.691 to 0.810 for Self-efficacy, and 0.758 to 0.833 for Ethical Awareness. All standardized factor loadings being above 0.50 and statistically significant (*p* < .001) indicate that each item adequately represents its latent variable [27]. The CFA path diagram is presented in Fig. 1.

### Item statistics, reliability, and convergent validity

Item-level statistics, internal consistency coefficients, composite reliability (CR), and average variance extracted (AVE) values for the final 20-item scale are presented in Table 4.

**Table 4:** Item analysis, reliability, construct reliability, and convergent validity results

Factor/Item	X̄	SD	rjx	α if deleted	λ (CFA)	α EFA	α CFA	CR	AVE
***Knowledge/Awareness***						***0.828***	***0.858***	***0.860***	***0.555***
M1	3.52	0.91	0.608	0.871	0.779				
M4	3.39	1.06	0.400	0.877	0.626				
M6	3.48	0.97	0.452	0.875	0.662				
M8	3.42	1.05	0.583	0.871	0.838				
M9	3.53	1.06	0.478	0.874	0.797				
***Willingness to Use AI***						***0.814***	***0.893***	***0.895***	***0.630***
M11	3.48	1.00	0.585	0.871	0.826				
M12	3.44	0.99	0.588	0.871	0.814				
M13	3.56	0.99	0.535	0.873	0.832				
M15	3.66	1.03	0.444	0.876	0.738				
M17	3.62	1.12	0.425	0.876	0.753				
***Self-efficacy***						***0.818***	***0.868***	***0.869***	***0.572***
M21	3.42	1.06	0.614	0.870	0.810				
M26	3.55	1.03	0.581	0.871	0.751				
M28	3.69	1.00	0.506	0.874	0.788				
M29	3.38	1.08	0.437	0.876	0.735				
M30	3.35	1.02	0.461	0.875	0.691				
***Ethical Awareness***						***0.801***	***0.902***	***0.904***	***0.652***
M31	3.69	1.25	0.386	0.878	0.758				
M35	3.99	1.03	0.362	0.878	0.826				
M38	3.92	1.06	0.361	0.878	0.798				
M39	3.94	1.07	0.456	0.875	0.822				
M40	3.97	1.10	0.498	0.874	0.833				
**Scale Total**						**0.880**	**0.911**		

Note. Means and standard deviations were calculated using the full sample (*N* = 416). Corrected item–total correlations (rjx) and Cronbach’s alpha if item deleted were computed from the EFA subsample (*n* = 200). Standardized factor loadings (λ), composite reliability (CR), and average variance extracted (AVE) were derived from the CFA subsample (*n* = 216). α EFA and α CFA denote subscale internal consistency coefficients calculated in the EFA and CFA subsamples, respectively

Table 4 shows that the corrected item–total correlations of all items ranged from 0.361 to 0.614, all exceeding the 0.30 cutoff. This indicates that each item is consistent with the measured construct [18].The finding that removal of any item did not increase the total Cronbach’s alpha (0.880) confirms that all items make meaningful contributions to the scale.

Standardized factor loadings (λ) from CFA ranged from 0.626 to 0.838, all above the 0.50 threshold. The highest factor loadings were observed for M8 (0.838) and M9 (0.797) in Knowledge/Awareness, M13 (0.832) and M11 (0.826) in Willingness to Use AI, M21 (0.810) and M28 (0.788) in Self-efficacy, and M40 (0.833) and M35 (0.826) in Ethical Awareness.

Regarding internal consistency reliability, subscale alpha values ranged from 0.801 to 0.828 in the EFA subsample (*n* = 200) and from 0.858 to 0.902 in the CFA subsample (*n* = 216), all exceeding the 0.70 threshold [30]. Composite reliability (CR) values ranged from 0.860 to 0.904, exceeding the 0.70 threshold. All AVE values (0.555–0.652) were above the 0.50 threshold, demonstrating that convergent validity was established [27].

### Discriminant validity

Discriminant validity was evaluated using the Fornell–Larcker criterion. According to this criterion, each subscale’s √AVE must exceed its correlations with other subscales [26]. Results are presented in Table 5.

**Table 5:** Inter-factor correlations and fornell–larcker discriminant validity criterion

	Knowledge/Awareness	Willingness to Use AI	Self-efficacy	Ethical Awareness
Knowledge/Awareness	**0.745**			
Willingness to Use AI	0.377	**0.794**		
Self-efficacy	0.575	0.689	**0.756**	
Ethical Awareness	0.261	0.376	0.385	**0.808**

Note. Diagonal values (bold): √AVE; below-diagonal values: inter-factor correlations from CFA

Table 5 shows that each subscale’s √AVE (diagonal values: 0.745–0.808) exceeded its correlations with other subscales (0.261–0.689). The √AVE of Knowledge/Awareness (0.745) exceeded its highest correlation with Self-efficacy (0.575). Similarly, the √AVE of Willingness to Use AI (0.794) exceeded its highest correlation with Self-efficacy (0.689). The Fornell–Larcker criterion being met across all comparisons confirms that the four subscales form distinguishable constructs and that the scale’s discriminant validity is supported [26].

Examination of inter-factor correlations revealed that the highest correlation was between Willingness to Use AI and Self-efficacy (0.689). This finding may be interpreted as students who are willing to use AI also feeling competent in using it. The moderate-to-high correlation between Knowledge/Awareness and Self-efficacy (0.575) indicates that AI knowledge is associated with self-efficacy perception. The lowest correlation being observed between Ethical Awareness and Knowledge/Awareness (0.261) suggests that ethical sensitivity is a relatively independent construct from knowledge level.

### Test–retest reliability

To evaluate the temporal stability of the scale, it was re-administered to 45 randomly selected participants from the EFA subsample after a two-week interval. Results are presented in Table 6.

**Table 6:** Test–retest reliability results (*n* = 45, 2-Week Interval)

Subscale	Test Mean ± SD	Retest Mean ± SD	*r*	ICC	t	*p*	Interpretation
Knowledge/Awareness	17.33 ± 4.29	17.27 ± 3.79	0.901	0.896	0.240	0.811	Good
Willingness to Use AI	17.84 ± 3.77	17.64 ± 3.90	0.910	0.910	0.822	0.415	Excellent
Self-efficacy	17.49 ± 4.54	17.44 ± 4.53	0.962	0.963	0.240	0.811	Excellent
Ethical Awareness	19.60 ± 4.63	19.42 ± 4.24	0.904	0.902	0.602	0.550	Excellent
**Total Scale**	**72.27 ± 12.69**	**71.78 ± 11.53**	**0.956**	**0.952**	**0.873**	**0.388**	**Excellent**

Note. ICC: Intraclass correlation coefficient (two-way random, single measures). Interpretation criteria (Koo & Li, 2016): <0.50 poor, 0.50–0.75 moderate, 0.75–0.90 good, > 0.90 excellent

Table 6 shows that ICC values for all subscales and the total scale ranged from 0.896 to 0.963. According to the classification proposed by Koo and Li (2016), the ICC value of Knowledge/Awareness (0.896) corresponds to “good” reliability, while Willingness to Use AI (0.910), Self-efficacy (0.963), Ethical Awareness (0.902), and the total scale (0.952) correspond to “excellent” reliability [28].

Pearson correlation coefficients ranged from 0.901 to 0.962, all statistically significant (*p* < .001), indicating strong consistency between the two measurements. Paired-samples t-test results showed no statistically significant difference between the first and second measurement means for any subscale or total score (all *p* > .05). The closeness of test and retest means (e.g., total scale: 72.27 vs. 71.78) confirms that the scale produced consistent measurements over the two-week period.

Taken together, these findings indicate that the NAIRS provides temporally stable and reliable measurements. The consistency of the scale when administered at different time points constitutes important evidence of measurement reliability.

### Summary of validity and reliability findings

When all findings regarding the psychometric properties of the Nursing Artificial Intelligence Readiness Scale (NAIRS) are considered together: (1) In terms of construct validity, the four-factor structure identified through EFA was confirmed by CFA, and all fit indices met acceptable or good fit criteria; (2) In terms of convergent validity, all CR (0.860–0.904) and AVE (0.555–0.652) values exceeded the threshold values; (3) In terms of discriminant validity, the Fornell–Larcker criterion was met across all comparisons; (4) In terms of internal consistency reliability, Cronbach’s alpha values exceeded acceptable levels in both the EFA and CFA subsamples; (5) In terms of test–retest reliability, ICC values indicated good to excellent stability. These findings demonstrate that the NAIRS is a valid and reliable instrument for assessing nursing students’ AI readiness levels.

## Discussion

In this study, the psychometric properties of the scale developed to assess nursing students’ readiness for artificial intelligence were examined, and the findings demonstrated that the instrument is valid and reliable. The increasing adoption of AI-based technologies in healthcare has made it increasingly important to evaluate healthcare professionals’ readiness, competencies, and perceptions regarding these technologies. AI-supported clinical decision support systems and data-driven care models are transforming healthcare delivery and increasing nurses’ interaction with these technologies [4, 31]. Therefore, determining nursing students’ readiness for artificial intelligence is important for developing nursing education to adapt to digital health environments [32].

The exploratory factor analysis conducted in this study revealed that the scale exhibited a four-factor structure explaining 52.08% of the total variance. Factor loadings ranging from 0.351 to 0.909 indicate that the items demonstrated adequate associations with their respective factors. In scale development studies, a total explained variance of 40–60% is generally considered acceptable [27]. In this regard, the explained variance ratio obtained in this study can be considered adequate. Moreover, the identification of multidimensional factor structures in scale development studies allows for a more comprehensive assessment of different dimensions of the measured construct [12]. Similar multidimensional factor structures have also been reported in scale development studies examining technology acceptance and digital health competencies in healthcare [33, 34]. From this perspective, the four-factor structure obtained in this study supports AI readiness being a multidimensional construct encompassing different dimensions such as knowledge/awareness, willingness to use AI, self-efficacy, and ethical awareness. The literature also reports that instruments developed for AI similarly exhibit multidimensional structures. For example, the Medical Artificial Intelligence Readiness Scale for Medical Students (MAIRS-MS) developed by Karaca et al. (2021) defined medical AI readiness as a multidimensional construct comprising cognitive, affective, and skill-based dimensions [9]. These findings indicate that the factor structure obtained in this study is consistent with the conceptual framework described in the literature.

The confirmation of the structure identified through EFA by CFA provides important evidence for the construct validity of the scale. In scale development studies, confirming the factor structure obtained through EFA with CFA is considered one of the fundamental methodological approaches for evaluating whether the theoretical structure of the instrument holds across different samples [16, 24]. The CFA results obtained in this study showed that the model fit indices were within acceptable limits. In particular, the CFI value of 0.971 and the RMSEA value of 0.044 demonstrate good fit between the proposed model and the data. In structural equation modeling studies, CFI and TLI values above 0.90 and RMSEA values below 0.08 and SRMR values below 0.10 are considered acceptable for model fit [23, 25].

When reliability analyses are examined, the internal consistency coefficients of the scale are high. Subscale Cronbach’s alpha coefficients ranged from 0.801 to 0.828 in the EFA subsample and from 0.858 to 0.902 in the CFA subsample, with the total scale alpha being 0.911. In the literature, a Cronbach’s alpha above 0.70 is considered adequate and above 0.80 is considered high internal consistency [27, 30]. In this regard, the obtained alpha values indicate that the scale has strong internal consistency. Furthermore, ICC values from test–retest analysis ranging from 0.896 to 0.963, with 0.952 for the total scale, demonstrate that the scale produces temporally stable measurements [28].

The four-factor structure observed in the present study is broadly consistent with prior work suggesting that readiness for medical AI is a multidimensional construct. In particular, the MAIRS-MS emphasized cognitive, skill-related, and attitudinal dimensions among medical students. The present study extends this line of work by proposing a nursing-specific structure that also explicitly incorporates ethical awareness, which is especially relevant in nursing because AI-supported decision-making in care settings raises questions related to patient safety, responsibility, confidentiality, and professional judgment.

The inclusion of an ethical awareness dimension is a notable contribution of the scale. In nursing education, readiness for AI should not be conceptualized solely as willingness to adopt technology or confidence in using it. Students are also expected to recognize the ethical implications of AI-assisted recommendations, data use, accountability, and the limits of automated systems in patient care.

Although a small number of items (e.g., M30 and M38) showed relatively lower factor loadings in the EFA solution, they were retained because they demonstrated acceptable corrected item–total correlations, satisfactory standardized CFA loadings, and meaningful contributions to content coverage within their respective domains. Their retention was guided not only by statistical criteria but also by theoretical considerations, as the removal of these items would have reduced the breadth of the self-efficacy and ethical awareness constructs represented in the scale. Nevertheless, future studies should continue to evaluate the performance of these items in different samples and consider potential refinement if consistently lower factor loadings are observed.

The inclusion of expert review and pilot testing during the development phase strengthened the content relevance, clarity, and comprehensibility of the items before psychometric evaluation.

The findings of this study have important implications for nursing education. The NAIRS may be used by nursing educators to identify strengths and gaps in students’ readiness for AI across the domains of knowledge, willingness to use AI, self-efficacy, and ethical awareness. The scale may also support curriculum development by helping educators evaluate the effectiveness of AI-related educational interventions and monitor changes in students’ readiness over time. As AI becomes increasingly integrated into clinical decision support systems, documentation processes, and digital health applications, nursing curricula should incorporate structured learning opportunities that address not only technical competencies but also ethical and professional aspects of AI use. Recent evidence on AI-supported applications, chatbots, and simulation in nursing education further supports the need for pedagogically structured, critically oriented AI learning experiences [4, 35, 36]. Accordingly, the assessment of nursing students’ AI readiness may provide an important evaluative framework for educational planning and research examining the integration of digital health technologies into nursing education. Unlike instruments adapted from other disciplines, the NAIRS was developed specifically for the nursing context and may serve as a practical tool for evaluating AI readiness within nursing education programs.

### Limitations

This study has several limitations that should be considered when interpreting the findings. First, the data were collected using self-report measures, which may be subject to response bias and social desirability effects. Second, the study employed a cross-sectional design, which does not allow for the evaluation of changes in AI readiness over time. Third, external criterion-related validity was not examined using an established comparison instrument, which limits the evidence for convergent validity with related constructs. Fourth, measurement invariance across sex, academic year, or prior AI-related training was not tested; therefore, the equivalence of the scale across subgroups remains to be established. Finally, the study was conducted at a single public university in Türkiye, which may limit the generalizability of the findings to nursing students from other institutions and educational settings. In addition, more than half of the participants were first-year students. Their limited clinical experience may have influenced their perceptions and understanding of the clinical applications of artificial intelligence. Future studies should evaluate the scale in more diverse samples with a more balanced distribution across academic years and educational settings.

## Conclusion and future directions

Taken together, these findings provide initial evidence that the NAIRS is a valid and reliable instrument for assessing nursing students’ readiness for artificial intelligence across four related but distinguishable domains: knowledge/awareness, willingness to use AI, self-efficacy, and ethical awareness. The scale may be useful for identifying educational needs, monitoring readiness profiles, and informing curriculum development in nursing education. Future studies should examine the scale in multicenter samples, test its measurement invariance across student subgroups, and explore its associations with related constructs such as AI literacy, AI anxiety, and technology acceptance.

## Supplementary Information

Below is the link to the electronic supplementary material.

Supplementary Material 1

## Data Availability

The datasets generated and/or analyzed during the current study are available from the corresponding author on reasonable request.
