# Supplementary material for: Development of the nursing artificial intelligence readiness scale for nursing students: a validity and reliability study
Source: BMC Nurs. 2026 Jul 16;25:961. doi: 10.1186/s12912-026-05054-4 (PMC13656420; doi:10.1186/s12912-026-05054-4)
Supplement: Supplementary file 1 [file 12912_2026_5054_MOESM1_ESM.docx]

**Additional file 1 (Supplementary File 1)**

**Nursing Artificial Intelligence Readiness Scale (NAIRS) — English Version**

**1. Description**

The Nursing Artificial Intelligence Readiness Scale (NAIRS) was developed by the authors for the present study to assess nursing students’ readiness for artificial intelligence (AI). It has not been previously published elsewhere. The final scale comprises 20 items across four subscales: Knowledge/Awareness (5 items), Willingness to Use AI (5 items), Self-efficacy (5 items), and Ethical Awareness (5 items).

The scale was originally developed and administered in Turkish. To support transparency and reuse by international readers, each item is presented below in both the original Turkish and an English translation.

**2. Response format**

Each item is rated on a 5-point Likert scale:

| **Response option (English)** | **Yanıt seçeneği (Turkish)** |
| --- | --- |
| 1 = Strongly Disagree | 1 = Kesinlikle Katılmıyorum |
| 2 = Disagree | 2 = Katılmıyorum |
| 3 = Undecided | 3 = Kararsızım |
| 4 = Agree | 4 = Katılıyorum |
| 5 = Strongly Agree | 5 = Kesinlikle Katılıyorum |

**3. Scale items**

| **No.** | **Item code*** | **Item (English)** | **Original item (Turkish)** |
| --- | --- | --- | --- |
| ***Subscale 1 — Knowledge/Awareness*** | | | |
| 1 | M1 | I believe I understand how artificial intelligence basically works. | Yapay zekanın temel işleyişini anladığımı düşünüyorum. |
| 2 | M4 | I know how artificial intelligence algorithms operate in decision-making processes. | Yapay zeka algoritmalarının karar verme süreçlerinde nasıl işlediğini biliyorum. |
| 3 | M6 | I know the contributions of artificial intelligence to the process of analyzing patient data. | Yapay zekanın hasta verilerini analiz etme sürecine katkılarını biliyorum. |
| 4 | M8 | I know how artificial intelligence technologies can improve patient outcomes. | Yapay zeka teknolojilerinin hasta sonuçlarını nasıl iyileştirebileceğini biliyorum. |
| 5 | M9 | I know the limitations of artificial intelligence in health care. | Yapay zekanın sağlık hizmetlerinde sınırlılıklarını biliyorum. |
| ***Subscale 2 — Willingness to Use AI*** | | | |
| 6 | M11 | I find using artificial intelligence in clinical practice useful. | Klinik uygulamalarda yapay zekayı kullanmayı faydalı buluyorum. |
| 7 | M12 | I am willing to use artificial intelligence for the health of my patients. | Hastalarımın sağlığı için yapay zekayı kullanmaya istekliyim. |
| 8 | M13 | I feel excited about working with artificial intelligence–supported applications. | Yapay zeka destekli uygulamalarda çalışmaktan heyecan duyuyorum. |
| 9 | M15 | I am ready to learn to use artificial intelligence technologies. | Yapay zeka teknolojilerini kullanmayı öğrenmeye hazırım. |
| 10 | M17 | I would prefer to use artificial intelligence–based patient monitoring systems. | Yapay zeka tabanlı hasta izleme sistemlerini kullanmayı tercih ederim. |
| ***Subscale 3 — Self-efficacy*** | | | |
| 11 | M21 | I believe I can use artificial intelligence systems confidently. | Yapay zeka sistemlerini güvenle kullanabileceğime inanıyorum. |
| 12 | M26 | I trust my knowledge and skills when making decisions related to artificial intelligence. | Yapay zeka ile ilgili kararlar alırken bilgi ve becerilerime güveniyorum. |
| 13 | M28 | I can integrate artificial intelligence technologies into the clinical decision-support process. | Yapay zeka teknolojilerini klinik karar destek sürecine entegre edebilirim. |
| 14 | M29 | I believe I can work independently in artificial intelligence–supported care practices. | Yapay zeka destekli bakım uygulamalarında bağımsız çalışabileceğime inanıyorum. |
| 15 | M30 | I can take responsibility in artificial intelligence–based care planning. | Yapay zeka tabanlı bakım planlamalarında sorumluluk alabilirim. |
| ***Subscale 4 — Ethical Awareness*** | | | |
| 16 | M31 | I think it is important for artificial intelligence to respect patient privacy. | Yapay zekanın hasta mahremiyetine saygı göstermesinin önemli olduğunu düşünüyorum. |
| 17 | M35 | Protecting patients’ rights during the use of artificial intelligence is important. | Yapay zeka kullanımı sırasında hasta haklarının korunması önemlidir. |
| 18 | M38 | I believe that artificial intelligence–supported treatment decisions should remain under human oversight. | Yapay zeka destekli tedavi kararlarının insan denetiminde olması gerektiğine inanıyorum. |
| 19 | M39 | I think that the use of artificial intelligence should respect human values. | Yapay zeka kullanımının insan değerlerine saygı göstermesi gerektiğini düşünüyorum. |
| 20 | M40 | I believe that artificial intelligence should be used not only to provide benefit but also with regard to ethical responsibilities. | Yapay zekanın sadece fayda sağlaması değil, etik sorumluluklar gözetilerek kullanılması gerektiğine inanıyorum. |

** “Item code” corresponds to the item labels used in the tables of the main manuscript (e.g., in the exploratory and confirmatory factor analysis tables). Items are numbered 1–20 in the order presented here.*

**4. Scoring**

Each item is scored from 1 to 5. Subscale scores are obtained by summing the item scores within each subscale (possible range for each subscale: 5–25). The total scale score is the sum of all 20 items and ranges from 20 to 100. Higher scores indicate greater readiness for artificial intelligence. No items are reverse-scored.
